# Comparison of the prognosis for different onset stage of cardiogenic shock secondary to ST-segment elevation myocardial infarction

**DOI:** 10.1186/s12872-020-01583-1

**Published:** 2020-06-19

**Authors:** Shuaihua Qiao, Jingmei Zhang, Zhenzhen Kong, Han Wu, Rong Gu, Hongyan Zheng, Biao Xu, Zhonghai Wei

**Affiliations:** 1 Department of Cardiology, Drum Tower Hospital, Medical School of Nanjing University, Nanjing, 210008 China; 2 Department of Cardiology, Yizheng Hospital, Nanjing Drum Tower Hospital Group, Yizheng, 211900 China

**Keywords:** Cardiogenic shock, Myocardial infarction, Percutaneous coronary intervention, Prognosis

## Abstract

**Objectives:**

The study was conducted to evaluate the outcomes of different onset stage of cardiogenic shock (CS) in the patients with ST-segment elevation myocardial infarction (STEMI).

**Methods:**

Total 675 STEMI patients who had undergone primary percutaneous coronary intervention (pPCI) from November 2010 to December 2017 in Nanjing Drum Tower Hospital were enrolled. According to the onset time of CS, the cohort was divided into three groups: Non-CS group, CS on admission group and Developed CS group. The short-term (30 days), middle-term (12 months) and long-term (80 months) outcomes were analyzed. COX proportional hazard models were established for identification of the predictors.

**Results:**

The all cause death, cardiac death and major adverse cardiac events (MACE) at 30 days were similar among the three groups. The incidence of MACE in the CS on admission group was significantly higher than the other two groups at 12 months. As to the long-term outcomes, the CS on admission group had lower survival rate than the other two groups. The Develop CS group had lower survival rate than Non-CS group numerically with a trend towards statistical significance. The incidence of cardiac death in the Non-CS group was the lowest. The incidence of MACE in the CS on admission group was much higher compared with the other two groups. After multivariate analysis, the independent predictors of all cause death included age, male sex, prior stroke and LVEF. The independent predictors of cardiac death included age, male sex, prior stroke, LVEF, CS on admission and developed CS. The independent predictors of MACE included age, prior stroke, LVEF, multivessel lesions, post-PCI TIMI grade 1 and CS on admission.

**Conclusions:**

The long-term outcomes of CS on admission group were the worst of all. The outcomes of Developed CS group laid between the other two groups. The consequences highlighted the importance of prevention for CS developing in the STEMI patients during hospitalization.

## Introduction

Cardiogenic shock (CS) is a fatal complication of acute myocardial infarction (AMI), occurring in 5 to 15% AMI cases [1–3]. Although CS has been declining slowly over the last decade due to the development of the therapeutic strategies [4], it has remained responsible for approximate 30–50% cardiac death in the AMI patients [5]. People have been making maneuvers to develop different mechanical circulatory support for improving the hemodynamic status in case of CS. To our disappointment, the efficacy in survival improvement is not significant according to the limited data [6–9]. On the contrary, a few studies suggested the intra-aortic balloon pump (IABP) could improve the prognosis in the patients without CS [10–12], which implied the mechanical circulatory support probably produce better efficacy in the patients with high likelihood of developing CS.

So far there is no definite criteria for identification of the subset patients. The previous studies have used various definitions, such as systolic blood pressure below 100 mmHg, heart rate above 100 beats per minute, Killips classification≥2, impaired left ventricular function, multivessel disease, etc [10, 12–14]. These different risk factors in the definitions are acquired from empirical experiences or regression analysis, which is also the leading cause of the heterogeneous results of the previous studies. Moreover, the prognosis of the AMI patients who actually develop CS in the real world is essential for assessing the value of prophylactic utility of mechanical circulatory support. Nonetheless, the relative studies and data on this facet are considerable limited. Thus, we perform the current study to compare the clinical outcomes of different onset time of CS in the patients with ST segment elevation myocardial infarction (STEMI) and evaluate the short and long-term prognosis of developed CS.

## Methods

### Study population

The diagnosis of STEMI was based on the criteria of American College of Cardiology/American Heart Association (ACC/AHA) [15] and the European Society of Cardiology [16]. Data was obtained from the databases in our institution and the ethics has been approved by the Medical Ethics Committee of Nanjing Drum Tower Hospital, Medical School of Nanjing University (2015–059-01).

The including criteria were as follows: (1) patients aged 18 ~ 90 years; (2) all patients presented to the emergency department of our hospital for AMI; (3) STEMI diagnosed by electrocardiography (ECG) in emergency department; (4) the patients were eligible for primary percutaneous coronary intervention (pPCI) and willing to receive the procedure.

The exclusion criteria were as follows: (1) the patients were younger than 18 years or older than 90 years old; (2) the patients did not receive emergency angiography; (3) the patients did not receive emergency revascularization after angiography; (4) the patients were suitable for emergency coronary artery bypass graft surgery (CABG); (5) the patients lost follow-up.

**Fig. 1:**
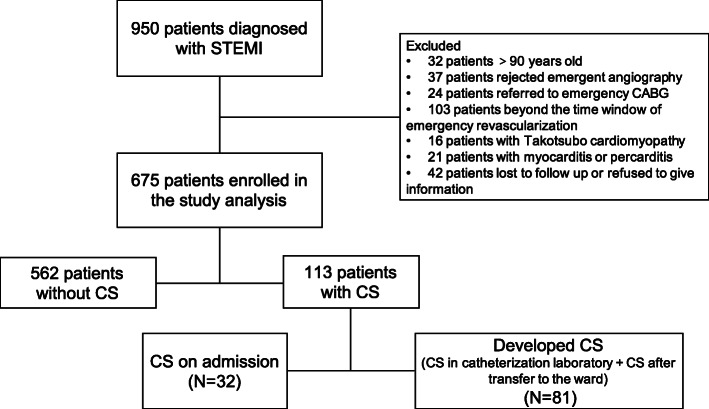
Flowchart of patient inclusion. STEMI: ST-segment elevation myocardial infarction; CS: cardiogenic shock; CABG: coronary artery bypass graft surgery

During the period from November 2010 to December 2017, total of 950 patients were diagnosed STEMI. The enrollment and exclusion procedure were shown in the flow chart (Fig. 1). Finally, 675 patients were enrolled in the study analysis. The patient cohort was divided into 3 groups based on the hemodynamic status and CS onset stage: 562 patients without CS during hospitalization (Non-CS group), 32 patients presenting CS when admitted in emergency department (CS on admission group) and 81 patients without CS on admission but developed CS during pPCI or after pPCI (Developed CS group).

### Study protocol

All the patients with acute chest pain in the emergency room accepted ECG within 10 min. STEMI was defined as new onset of ST segment elevation at the J point in at least 2 contiguous leads of above 2 mm in men or above 1.5 mm in women at V2 and V3 lead and/or of above 1 mm in other leads. The new onset left bundle branch block was regarded equivalent to STEMI [17]. After taking loading dose of dual antiplatelet drugs (aspirin 300 mg and ticagrelor 180 mg/clopidogrel 600 mg), patients were immediately transferred to the catheterization laboratory (Cath Lab) for emergency coronary angiography. Heparin was administered at a dose of 70–100 IU/kg, while tirofiban, urokinase or argatroban were used if necessary. Revascularization strategy was individualized according to the angiography results. CS was defined as systolic blood pressure < 90 mmHg for > 30 min, the need for infusion of inotropic agents to maintain adequate blood pressure or clinical signs of pulmonary congestion and end-organ hypoperfusion. If the STEMI patients were likely to develop CS during pPCI, whether the prophylactic IABP was deployed depended on the judgment of the interventionists. If the patients had already experienced hemodynamic instability or CS after admission, the rescue IABP and/or vasoactive agents were administrated also at the discretion of the interventionists. All the procedures were accomplished by experienced and qualified interventionists.

### Follow up

The patients were followed up via telephone or clinics. The follow-up was carried out periodically until date of death, or 30 October 2018. Endpoints include all cause death and major adverse cardiac events (MACE) at 30 days, 12 months and 80 months. MACE was defined as composite of cardiac death, recurrent myocardial infarction (MI) or angina, non-fatal stroke and the re-hospitalization due to worsening of cardiac function. The cardiac death was defined as the deaths due to cardiac diseases such as myocardial infarction, arrhythmia, heart failure and any death that was not clearly non-cardiac.

### Statistical analysis

The continuous variables were described as the mean ± standard deviations (SD) when they were normally distributed or median and interquartile range (IQR) when they were skewed. The categorical variables were presented as frequency and percentages. The comparisons of continuous variables among the three groups were used by one-way ANOVA or Kruskal-Wallis test. The comparisons of categorical variables among the groups are used by χ^2^ test or Fisher test. Survival analysis and cumulative incidence of MACE was assessed by Kaplan-Meier plot and Log rank test. Univariate and multivariate COX proportional hazard model are established for identification of the independent predictors. *P* value < 0.05 is considered as statistical significance and intervals (CI) of 95% is applied in the overall cohort. The statistical analysis was performed by SPSS 17.0 (SPSS Inc., Chicago, Ill., USA) and STATA 12.0 (StataCop., College Station, Texas, USA).

## Result

### Characteristics of study cohort

Total 675 patients were enrolled in this study cohort. The mean age of the patients was 64 ± 13 years and 79% were male. Based on the different onset stage of CS, 32 patients (5.6%) presented CS on admission and 81 patients (14.1%) developed CS later during hospitalization (in Cath Lab or CCU). The rest patients had no CS (Table 1).

**Table 1:** Characteristics of the study cohort

	Non-CS (*n* = 562)	CS on admission (*n* = 32)	Developed CS (*n* = 81)	*p-*value
Age (years), mean ± SD	64 ± 12.7	66 ± 11.7	64 ± 12.7	0.593
Male sex, n (%)	444 (79)	25 (78)	64 (79)	0.993
Hypertension, n (%)	359 (64)	16 (50)	44 (54)	0.089
Diabetes, n (%)	142 (25)	14 (44)	13 (16)	0.009^#, &&^
Smoking, n (%)	315 (56)	17 (53)	46 (57)	0.938
Prior stroke, n (%)	76 (14)	7 (22)	10 (12)	0.383
Hyperlipidaemia, n (%)	44 (8)	5 (16)	5 (6)	0.233
Multivessel lesions, n (%)	140 (25)	13 (41)	32 (40)	0.005^#, **^
Known kidney dysfunction, n (%)	67 (12)	8 (25)	12 (15)	0.086
Prior MI, n (%)	15 (3)	3 (9)	2 (2)	0.09
Pre-procedural SBP (mmHg), mean ± SD	125 ± 18.9	80 ± 6.6	112 ± 20.6	< 0.001^###, ***, &&&^
Pre-procedural HR (bpm), mean ± SD	79 ± 14.7	114 ± 18.5	87 ± 16.5	< 0.001^###, ***, &&&^
Shock index, mean ± SD	0.65 ± 0.16	0.96 ± 0.22	0.81 ± 0.25	< 0.001^###, ***, &&&^
LVEF(%), mean ± SD	45 ± 5.6	44.6.4	45 ± 6.6	0.319
Creatinine (mmol/L), mean ± SD	70(24)	76 (39)	71 (24)	0.409
TG (mmol/L), mean ± SD	1.66 ± 1.09	1.39 + ±0.73	1.61 ± 0.88	0.377
TCh (mmol/L), mean ± SD	4.29 ± 1.02	4.27 ± 1.19	4.27 ± 0.97	0.985
LDL-C (mmol/L), mean ± SD	2.38 ± 0.74	2.46 ± 0.84	2.41 ± 0.70	0.792
HDL-C (mmol/L), mean ± SD	1.00 ± 0.38	1.00 ± 0.28	0.95 ± 0.34	0.525
Symptom-wire interval (min),median (IQR)	240 (328)	168(202)	237 (242)	0.067
IABP utility, n (%)	15 (3)	8 (25)	21 (26)	< 0.001^###, ***^
Anterior MI, n (%)	275 (49)	10 (31)	29 (36)	0.018^#,*^
Killips classification				< 0.001^###,*,&&&^
I, n (%)	463 (82)	0	62 (77)	
II, n (%)	88 (16)	0	13 (16)	
III, n (%)	12 (2)	0	6 (7)	
V, n (%)	0	32 (100)	0	
Post-procedure TIMI flow				0.079
3, n (%)	557 (99)	31 (97)	78 (96)	
2, n (%)	4 (1)	0 (0)	3 (4)	
1, n (%)	1 (0)	1 (3)	0 (0)	
IRA				< 0.001^##, *^
LM/LM-LAD, n (%)	286 (51)	10 (31)	29 (36)	
LCX/OM, n (%)	90 (16)	2 (6)	11 (13)	
RCA/PDA/PL, n (%)	186 (33)	20 (63)	41 (51)	
Medicine				
β blocker, n (%)	483 (86)	21 (67)	63 (78)	0.177
ACEI/ARB, n (%)	427 (76)	21 (67)	54 (67)	0.517
Aldactone, n (%)	90 (16)	11 (33)	15 (18)	0.427

*SBP* Stytolic blood pressure, *HR* Heart rate, *bpm* beats per minute, *SI* Shock index, *LVEF* Left ventricular ejection fraction, *TG* Triglyceride, *LDL-C* Low density lipoprotein cholesterol, *HDL-C* High density lipoprotein cholesterol, *TCh* Total cholesterol, *IABP* Intra-aortic balloon pump;MI: myocardial infarction, *TIMI* Thrombolysis in myocardial infarction, *IRA* Infarct-related artery, *LM* Left main artery, *LAD* Left anterior descending branch, *LCX* Left circumflex branch, *OM* Obtuse marginal branch, *RCA* Right coronary artery, *PDA* Posterior descending artery, *PL* Posterior branch of left ventricle, *ACEI* Angitensin conveting enzyme inhibitor, *ARB* Angiotensin receptor blocker

# *P* < 0.05, ##*P* < 0.01, ### *P* < 0.001: Non-CS group vs CS on admission group

* *P* < 0.05,**P < 0.01,*** *P* < 0.001: Non-CS group vs Developed CS group

& *P* < 0.05,&&*P* < 0.01, &&& *P* < 0.001: CS on admission group vs Developed CS group

CS on admission group had higher percentage of diabetes than Non-CS group and Developed CS group (*P* = 0.021 and *P* = 0.002, respectively). The pre-procedural systolic blood pressure (SBP) and pre-procedural heart rate (HR) in CS on admission group were both significantly different from the other two groups (*P* < 0.001 for both). Correspondingly, the shock index (SI) in the CS on admission group was therefore the highest. There were more patients had left main artery (LM)/ left anterior descending branch (LAD) as culprit in Non-CS group whereas more patients had right coronary artery (RCA) as culprit in CS on admission group (*P* < 0.001). The patients in Developed CS group had higher proportion of Killips II/III than the patients in Non-CS group (Table 1).

### Endpoints follow-up

The patient cohort were followed up for median 36 months (IQR: 23 ~ 59 months). There were total 78 patients (11.6%) experiencing death of different causes. Among them, 18 patients died from non-cardiac origins diseases (multi-organs failure: 8 patients, tumor: 5 patients, infection of lungs: 2 patients, end stage of renal dysfunction: 1 patient, trauma: 2 patients).

The all cause death and MACE at 30 days were shown in the Table 2. There was only higher incidence of non-fatal stroke in the CS on admission group in spite of the quite small frequency. The other endpoints were similar among the three groups.

**Table 2:** Endpoint events in the study cohort at 30 days

Events	Non-CS (***n*** = 562)	CS on admission (***n*** = 32)	Developed CS (***n*** = 81)	***p-***value
All cause death, n (%)	13(2)	1(3)	2(2)	0.956
MACE				
Cardiac death, n (%)	13(2)	1(3)	2(2)	0.956
Angina/Recurrent MI, n (%)	4(1)	0(0)	0(0)	0.667
Worsening of cardiac function, n (%)	2(0)	1(3)	1(1)	0.101
Non-fatal stroke, n (%)	0(0)	1(3)	0(0)	*P* < 0.001

*MI* Myocardial infarction, *MACE* Major adverse cardiac events

**Fig. 2:**
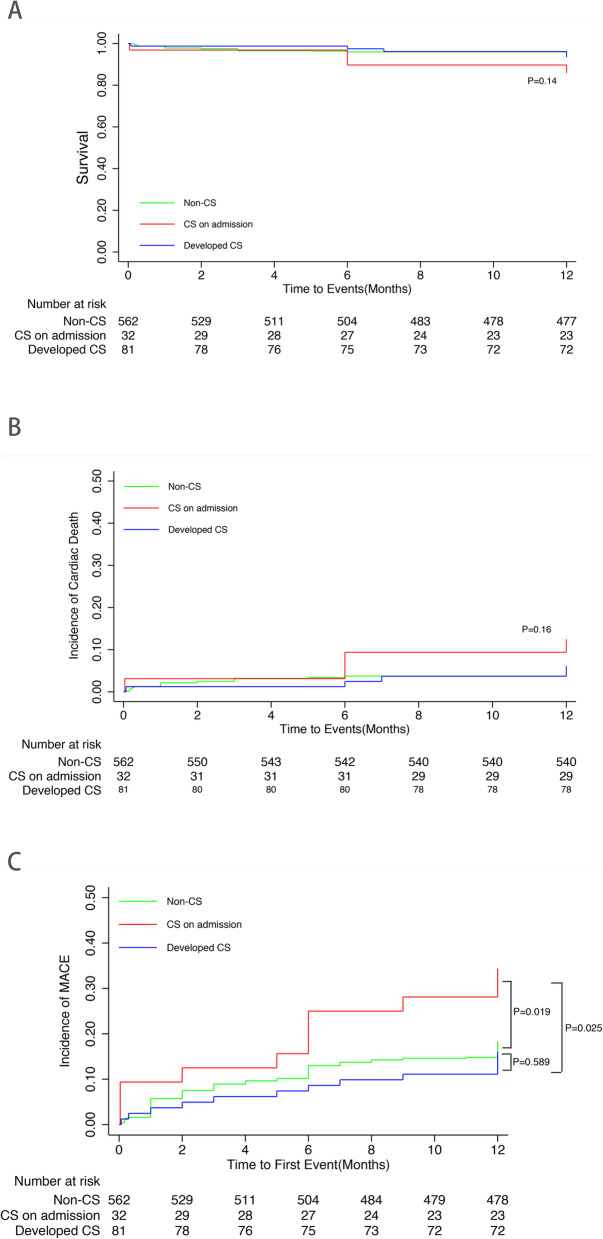
The outcomes of the study cohort at 12 months. **a**. survival curves. **b**. cardiac death curves. **c**. MACE curves. The incidence of MACE at 12 months in the CS on admission group was significantly higher than the other two groups. On the contrary, there were no differences in the all cause death and cardiac death at 12 months among the three groups MACE: major adverse cardiac events

**Fig. 3:**
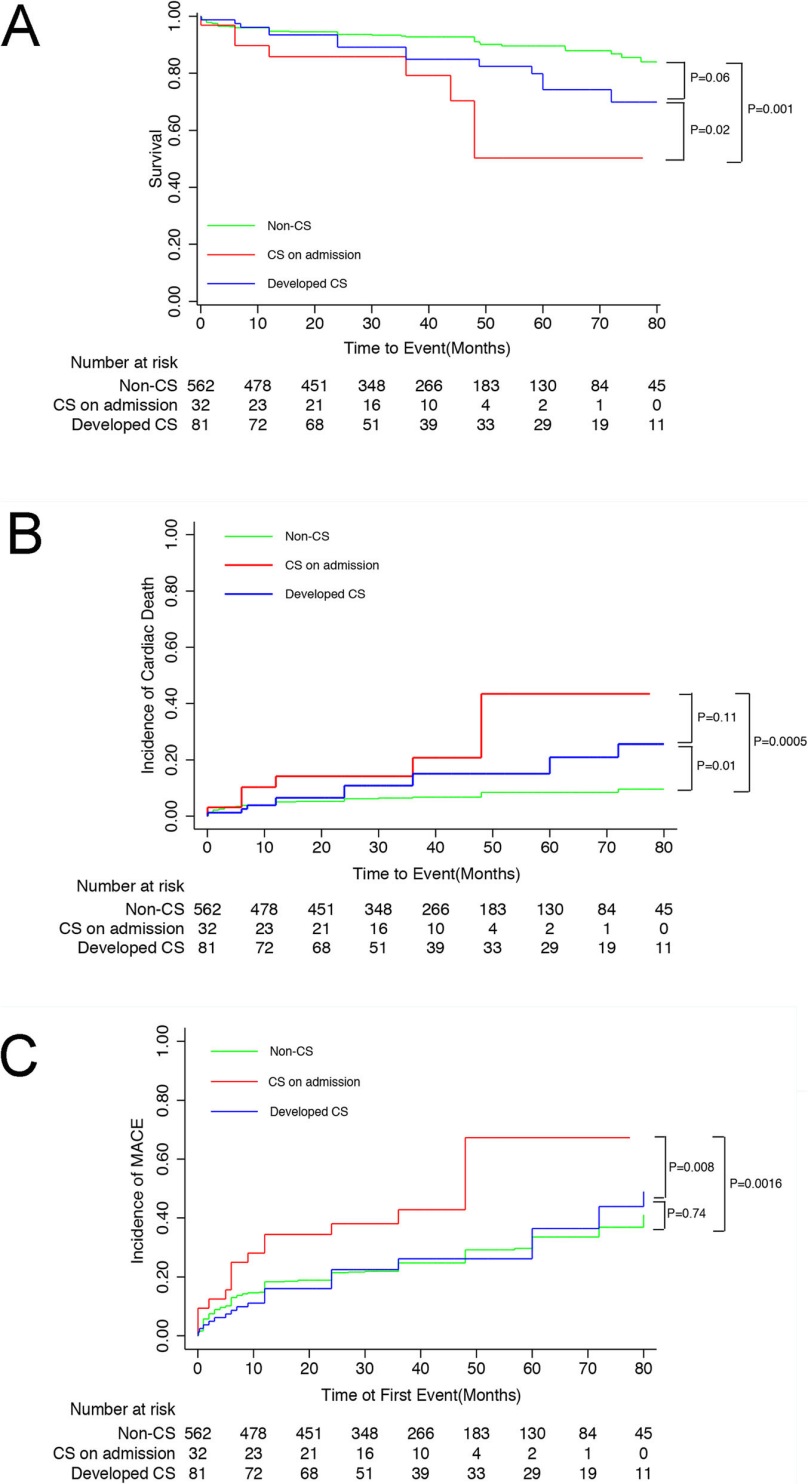
The long-term outcomes of the study cohort. **a**. survival curves. **b**. cardiac death curves. **c**. MACE curves. We found the CS on admission group had much lower survival rate and higher incidence of cardiac death and MACE than the other two groups. CS: cardiogenic shock; MACE: major adverse cardiac events

The middle-term outcomes were exhibited in Fig. 2. At 12 months, the curve of all cause death and cardiac death did not separate (Fig. 2a and b). However, the incidence of MACE in CS on admission group had already been significantly higher than the other groups (Fig. 2c). With regard to the long-term outcomes, the CS on admission group had the lowest survival rate among the three groups whereas Develop CS group had lower survival rate than Non-CS group numerically with a trend towards statistical significance (Fig. 3a). Furthermore, the incidence of cardiac death in the Non-CS group was the lowest, while the cardiac death rate was not different between CS on admission group and Developed CS group (Fig. 3b). The incidence of MACE in the CS on admission group was much higher compared with the other two groups. But the MACE in the Non-CS group and Developed CS group were not significantly different (Fig. 3c).

COX proportional hazard models were established for all cause death, cardiac death and MACE to adjust the confounding factors and identify the independent predictors of the long-term outcomes. There were total 24 variables participating the univariate analysis including 10 continuous variables and 14 categorical variables. After univariate analysis, the covariates with *P* < 0.1 and the covariates with *P* > 0.1 but with clinical significance were extracted for multivariate regression analysis. The variables were selected with likelihood ratio test and the covariates with *P* < 0.05 were considered statistically significant.

After univariate and multivariate analysis, there were four covariates as independent predictors of all cause death: age, male sex, prior stroke and left ventricular ejection fraction (LVEF) (Table 3). As to the cardiac death, the following covariates were identified as independent predictors: age, male sex, prior stroke, LVEF, CS on admission and developed CS (Table 4). With regard to the MACE, six covariates were identified as independent predictors: age, prior stroke, LVEF, multivessel lesions, post-PCI TIMI grade 1 and CS on admission (Table 5).

**Table 3:** COX proportional hazard model for the all cause death of the study cohort

	Univariate			Multivariable		
	HR	95% CI	*p*-value	HR	95% CI	*p*-value
Age	1.094	1.070–1.119	< 0.001	1.071	1.045–1.099	< 0.001
Male sex	3.383	2.167–5.282	< 0.001	2.105	1.292–3.431	0.003
Hypertension	1.13	0.709–1.802	0.608	–	–	–
Diabetes	1.193	0.733–1.941	0.478	–	–	–
Smoking	0.753	0.353–1.165	0.135	–	–	–
Prior Stroke	3.749	2.348–5.985	< 0.001	2.209	1.347–3.621	0.002
Hyperlipdemia	0.279	0.069–1.137	0.075	–	–	–
Known kidney dysfunction	1.663	1.042–2.652	0.033	–	–	–
Prior MI	2.138	0.780–5.865	0.14	–	–	–
Pre-procedural SBP	0.988	0.977–0.998	0.023	–	–	–
Pre-procedural HR	1.021	1.009–1.033	0.001	–	–	–
LVEF, per % increased	0.9	0.868–0.933	< 0.001	0.931	0.893–0.967	< 0.001
TG, per mmol/L increased	0.72	0.565–1.003	0.056	–	–	–
TCh, per mmol/L increased	0.965	0.769–1.209	0.755	–	–	–
LDL-C, per mmol/L increased	0.872	0.634–1.199	0.4	–	–	–
HDL-C, per mmol/L increased	1.61	0.712–2.553	0.079	–	–	–
Multivessel lesions	1.778	1.148–2.754	0.01	–	–	–
Time of symptom-wire, per hour increased	1.016	1.008–1.024	< 0.001	–	–	–
IABP utility	1.449	0.697–3.014	0.32	–	–	–
Shock index (per 0.1 increased)	1.152	1.078–1.230	< 0.001	–	–	–
Anterior MI	0.726	0.465–1.132	0.158	–	–	–
Onset stage of CS						
Non- CS (reference)	1	–	–	–	–	–
CS on admission	3.412	1.619–7.191	0.001	–	–	–
developed CS	1.692	0.954–2.999	0.072	–	–	–
Killips Classification						
Killips = 1(reference)	1	–	–	–	–	–
Killips = 2	2.405	1.426–4.055	0.001	–	–	–
Killips = 3	3.774	1.493–9.538	0.005	–	–	–
Killips = 4	4.566	2.131–7.782	< 0.001	–	–	–
post-PCI TIMI flow of IRA						
3(reference)	1	–	–	–	–	–
2	1.651	0.229–11.898	0.619	–	–	–
1	4.762	0.658–34.465	0.122	–	–	–

*CS* Cardiogenic shock, *SBP* Stytolic blood pressure, *HR* Heart rate, *bpm* beats per minute, *LVEF* Left ventricular ejection fraction, *TG* Triglyceride, *LDL-C* Low density lipoprotein cholesterol, *HDL-C* High density lipoprotein cholesterol, *TC* Total cholesterol, *IABP* Intra-aortic balloon pump, *MI* Myocardial infarction, *TIMI* Thrombolysis in myocardial infarction, *IRA* Infarct-related artery, *PCI* percutaneous coronary intervention

**Table 4:** COX proportional hazard model for the cardiac death of the study cohort

	Univariate			Multivariate		
	HR	95% CI	*p*-value	HR	95% CI	*p*-value
Age	1.066	1.041–1.091	< 0.001	1.068	1.038–1.099	< 0.001
Male sex	3.543	2.132–5.889	< 0.001	2.100	1.196–3.688	0.01
Hypertension	1.968	1.082–3.582	0.027	–	–	–
Diabetes	1.429	0.835–2.444	0.193	–	–	–
Smoking	0.684	0.288–1.011	0.099	–	–	–
Prior Stroke	1.865	1.007–3.453	0.047	1.989	1.124–3.523	0.018
Hyperlipdemia	0.177	0.024–1.276	0.086	–	–	–
Known kidney dysfunction	1.764	1.035–3.007	0.037	–	–	–
Prior MI	1.345	0.328–5.519	0.681	–	–	–
Pre-procedural SBP	0.992	0.975–1.001	0.081	–	–	–
Pre-procedural HR	1.019	1.003–1.035	0.017	–	–	–
LVEF, per % increased	0.949	0.909–0.991	0.017	0.911	0.871–0.954	< 0.001
TG, per mmol/L increased	0.889	0.679–1.165	0.394	–	–	–
TCh, per mmol/L increased	0.811	0.623–1.055	0.118	–	–	–
LDL-C, per mmol/L increased	0.74	0.518–1.057	0.098			
HDL-C, per mmol/L increased	1.075	0.572–2.020	0.823	–	–	–
Multivessel lesions	1.954	1.191–3.208	0.008	–	–	–
Time of symptom-wire, per hour increased	1.011	0.999–1.023	0.081	–	–	–
IABP utility	2.004	0.951–4.224	0.068	–	–	–
Shock index (per 0.1 increased)	1.2	1.079–1.334	0.001	–	–	–
Anterior MI	0.795	0.479–1.319	0.374	–	–	–
Onset stage of CS						
Non- CS (reference)	1	–	–	1	–	–
CS on admission	3.345	1.496–7.479	0.003	2.739	1.210–6.204	0.016
developed CS	2.346	1.252–4.395	0.008	2.233	1.164–4.286	0.016
Killips Classification						
Killips = 1(reference)	1	–	–	–	–	–
Killips = 2	2.737	1.502–4.988	0.001	–	–	–
Killips = 3	3.84	1.694–8.700	0.007	–	–	–
Killips = 4	4.13	1.460–11.681	0.001	–	–	–
post-PCI TIMI flow of IRA						
3(reference)	1	–	–	–	–	–
2	4.403	1.071–18.098	0.04	–	–	–
1	6.488	1.191–27.228	0.035	–	–	–

*MACE* Major adverse cardiac events, *CS* Cardiogenic shock, *SBP* Stytolic blood pressure, *HR* Heart rate, *bpm* beats per minute, *LVEF* Left ventricular ejection fraction, *TG* Triglyceride, *LDL-C* Low density lipoprotein cholesterol, *HDL-C* High density lipoprotein cholesterol, *TC* Total cholesterol, *IABP* Intra-aortic balloon pump, *MI* Myocardial infarction, *TIMI* Thrombolysis in myocardial infarction, *IRA* Infarct-related artery, *PCI* Percutaneous coronary intervention

**Table 5:** COX proportional hazard model for the MACE of the study cohort

	Univariate			Multivariate		
	HR	95% CI	*p*-value	HR	95% CI	*p*-value
Age	1.028	1.016–1.040	< 0.001	1.017	1.005–1.031	0.005
Male sex	1.413	1.010–1.882	0.043	–	–	–
Hypertension	1.009	0.760–1.340	0.95	–	–	–
Diabetes	1.23	0.912–1.660	0.175	–	–	–
Smoking	0.812	0.617–1.068	0.136	–	–	–
Prior Stroke	1.863	1.338–2.594	< 0.001	1.639	1.146–2.344	0.007
Hyperlipdemia	0.909	0.545–1.514	0.713	–	–	–
Known kidney dysfunction	0.827	0.589–1.162	0.274	–	–	–
Prior MI	1.549	0.763–3.145	0.226	–	–	–
Pre-procedural SBP	0.996	0.990–1.002	0.213	–	–	–
Pre-procedural HR	1.005	0.997–1.013	0.192	–	–	–
LVEF, per % increased	0.962	0.940–0.984	0.001	0.978	0.954–0.997	0.044
TG, per mmol/L increased	0.892	0.770–1.032	0.124	–	–	–
TCh, per mmol/L increased	0.927	0.807–1.065	0.285	–	–	–
LDL-C, per mmol/L increased	0.989	0.933–1.048	0.700	–	–	–
HDL-C, per mmol/L increased	0.827	0.556–1.230	0.348	–	–	–
Multivessel lesions	1.444	1.091–1.911	0.01	1.462	1.098–1.947	0.009
Time of symptom-wire, per hour increased	1.003	0.994–1.013	0.477	–	–	–
IABP utility	1.108	0.665–1.847	0.693	–	–	–
Shock index (per 0.1 increased)	1.051	1.003–1.102	0.038	–	–	–
Anterior MI	0.739	0.562–0.972	0.031	–	–	–
Onset stage of CS						
Non- CS (reference)	1	–	–	1	–	–
CS on admission	2.252	1.345–3.770	0.002	1.948	1.164–3.261	0.011
developed CS	1.069	0.711–1.608	0.748	1.082	0.691–1.691	0.73
Killips Classification						
Killips = 1(reference)	1	–	–	–	–	–
Killips = 2	1.34	0.933–1.925	0.113	–	–	–
Killips = 3	1.864	0.914–3.801	0.087	–	–	–
Killips = 4	2.389	1.423–4.013	0.001	–	–	–
post-PCI TIMI flow of IRA						
3 (reference)	1	–	–	1	–	–
2	2.528	0.938–6.812	0.067	2.604	0.634–10.078	0.181
1	3.38	0.837–13.643	0.087	3.108	1.130–8.026	0.02

*MACE* Major adverse cardiac events, *CS* Cardiogenic shock, *SBP* Stytolic blood pressure, *HR* Heart rate, *bpm* beats per minute, *LVEF* Left ventricular ejection fraction, *TG* Triglyceride, *LDL-C* Low density lipoprotein cholesterol, *HDL-C* High density lipoprotein cholesterol, *TC* Total cholesterol, *IABP* Intra-aortic balloon pump, *MI* Myocardial infarction, *TIMI* Thrombolysis in myocardial infarction, *IRA* Infarct-related artery, *PCI* Percutaneous coronary intervention

## Discussion

The incidence of CS secondary to STEMI varies from 4.0 to 6.2% in Chinese patients cohort [18, 19], which is quite similar to the western countries [2, 20]. It has been reported that time of revascularization is a predictor of the survival at 1 year [21]. Nonetheless, the mortality of CS is still up to 50% so far according to the previous data [5, 22–24], although the timely reperfusion has been extensively performed worldwide for decades.

Total 113 patients in our study cohort presented CS after STEMI occurrence, accounting for around 16.7%. Thirty-two patients of them had been in CS status when admission while the other 81 patients developed CS during pPCI or after pPCI, which was quite similar to the previous findings [25, 26]. Hence, it is necessary to pay more attention to the likelihood of progressive or sudden exacerbation of hemodynamical status. One of the most important cause responsible for CS developed during hospitalization is reperfusion injury (RI) as it is associated with approximate 50% of the infarct size [27]. That could explain that most of the patients with developed CS present hemodynamical compromising after restoration of coronary blood flow in the real world. Some factors were identified as predictors of developed CS, including age, SBP, HR, SI, diabetes, LVEF, Killips classification, etc [26, 28, 29]. However, there were few studies evaluating the impact of the different onset stage of CS on the long-term prognosis.

In the current study, we found the incidence of all cause death, cardiac death and MACE at 30 days were all similar among the three groups. On the contrary, Laust Obling et al. revealed the CS patients had significant lower survival rate than non-CS patients at 30 days, while the patients with different time onset of CS had similar survival rate [28]. The constitution of infarct location is probably the pivotal factor which makes the significant difference. In the Obling’s study, anterior AMI accounted for 65% in the late CS group and 43% in CS on admission group. In contrast, anterior AMI only accounted for 31% in the CS on admission group and 35% in Developed CS group in our cohort, while inferior/posterior AMI totally accounted for nearly 55% in CS-containing groups (CS on admission +Developed CS). Thus, the current cohort had much better short-term prognosis than Obling’s study cohort. Moreover, LM occlusion mostly manifested widely depressed ST segment on ECG, which were not included in our study cohort. The short-term mortality of the current cohort was thereby much lower.

With regard to the long-term outcomes, patients in the CS on admission group had the worst prognosis no matter in all cause death, cardiac death or MACE, whereas the patients in Non-CS group had the best results in contrast. Of note, the Developed CS group had a higher survival rate than CS on admission group and had a trend towards a lower survival rate than the Non-CS group. Similarly, the incidence of cardiac death and MACE in the Developed CS group also laid between the other two groups. A previous study has reported that some predictors including age, estimated glomerular filtrated rate, LVEF, SI were related to the 5-year MACE in AMI patients [30]. In the current cohort, the influences of the different onset stage of CS on the long-term outcomes were the main concerns. Thus, we introduced this variable into the multivariate model. After adjustment of the confounding factors, the different onset stage of CS was not an independent predictor of long-term all cause death. As to all cause death, there was also death of other origins including multi-organs failure, cancer, pulmonary disease, which were more associated with the basic conditions of the patients rather than shock. Nevertheless, CS on admission and developed CS were both independent predictors of cardiac death, while only CS on admission was the independent predictor of MACE. It is interesting that developed CS could cause higher cumulative incidence of cardiac death in comparison with Non-CS group, whereas the MACE was similar between the two groups. It was thought partly associated with the different constitution between the two groups. Developed CS group had higher proportion of inferior/posterior infarction and lower proportion of anterior infarction than Non-CS group. Anterior infarction is more likely to cause left ventricular remodeling and recurrent heart failure during the long-term follow-up.

Our findings had indicated that the onset stage of CS had an impact on the long-term prognosis. A study performed by Giuseppe et al. revealed that the mortality in the patients with developed CS was numerically higher than the patients with CS on admission but without statistical significance [26]. In that study, they just focused on the in-hospitalization death, not reporting the long-term death. Besides, the cohort in his study also included the patients without revascularization, which was quite different from our patient cohort. Another study carried out by Lindholm et al. demonstrated that the different onset time of CS after AMI occurrence could produce significantly different outcomes [31]. Early CS showed better prognosis than late CS no matter in short-term and long-term mortality, which seemed to be opposite to the current study. However, it should be noticed that the group division criteria were set different from our study. Moreover, the patient cohort in Lindholm’s study enrolled both STEMI and non-ST segment elevation myocardial infarction (NSTEMI). NSTEMI usually causes late developed CS and leads to much higher mortality [32–34], which was probably another cause responsible for the different outcomes between the two studies.

In our study, the results coming from the real data illustrated two clinical impacts. First, CS due to STEMI definitely produces compromising long-term outcomes. The revascularization therapy should be carried out as early as possible. Second, it is important and necessary to prevent the developed CS in the patients with STEMI. The interventionists have been made lots of maneuver to figure out the strategies for preventing the developed CS, particularly due to the reperfusion injury. Mechanical circulatory supports are usually the preferable choice for this purpose. The useful predictors of developed CS has been established in lots of previous studies [28, 29, 32]. However, it is a practical task that how to distinguish the patients with impending CS using the various predictors. There has been only a handful of studies providing the criteria for high-risk patients [10, 13, 35]. Nonetheless, the definitions were not consistent with each other. Furthermore, the conclusions of the benefits from prophylactic use of mechanical circulatory support in the AMI patients with high risk of CS were largely acquired from observational or retrospective studies. Thus, more randomized, controlled, prospective clinical trials are necessary to confirm the value of prevention of the developed CS in STEMI patients.

## Limitations

The current study has several limitations. (1) It is an observational study, which has the intrinsic shortcomings. The biases are unable to be avoided completely despite of the adjustment of confounding factors using regression analysis. (2) The CS caused by LM occlusion usually presents non-ST segment elevation and was not included in the current study. Consequently, the mortality of the patients presenting CS on admission was probably underestimated. (3) There were considerable STEMI patients were excluded from the current cohort due to accepting elective PCI, which accounted for approximate 19% of the total STEMI patients. It could possibly influence the assessment of the outcomes of the STEMI patients in the real world.

## Data Availability

The information and data of the study population were extracted from Hospital Information System and were recorded manually in EXCEL to form the database. Thus, our database is not an online database and it is not open to public because the individual privacy of the participants should be protected.

## Abbreviations

ACEI: Angiotensin converting enzyme inhibitor
AMI: Acute myocardial infarction
ARB: Angiotensin receptor blocker
CABG: Coronary artery bypass graft
CCU: Cardiac care unit
CS: Cardiogenic shock
HDL-C: High density lipoprotein cholesterol
HR: Heart rate
IABP: Intra-aortic balloon pump
IRA: Infarct-related artery
LAD: Left anterior descending branch
LCX: Left circumflex branch
LDL-C: Low density lipoprotein cholesterol
LM: Left main artery
LVEF: Left ventricular ejection fraction
MACE: Major adverse cardiac events
NSTEMI: Non-ST segment elevation myocardial infarction
OM: Obtuse marginal branch
PDA: Posterior descending artery
PL: Posterior branch of left ventricle
pPCI: Primary percutaneous coronary intervention
RCA: Right coronary artery
RI: Reperfusion injury
SBP: Systolic blood pressure
SI: Shock index
STEMI: ST segment elevation myocardial infarction
TCh: Total cholesterol
TG: Triglyceride
TIMI: Thrombolysis in myocardial infarction

## References

[1] Aissaoui N, Puymirat E, Tabone X, Charbonnier B, Schiele F, Lefevre T, Durand E, Blanchard D, Simon T, Cambou JP, Danchin N (2012). Improved outcome of cardiogenic shock at the acute stage of myocardial infarction: a report from the usik 1995, usic 2000, and fast-mi french nationwide registries. Eur Heart J.

[2] Goldberg RJ, Spencer FA, Gore JM, Lessard D, Yarzebski J (2009). Thirty-year trends (1975 to 2005) in the magnitude of, management of, and hospital death rates associated with cardiogenic shock in patients with acute myocardial infarction: a population-based perspective. Circulation..

[3] Jeger RV, Radovanovic D, Hunziker PR, Pfisterer ME, Stauffer JC, Erne P, Urban P (2008). Ten-year trends in the incidence and treatment of cardiogenic shock. Ann Intern Med.

[4] Movahed MR, Khan MF, Hashemzadeh M (2015). Age adjusted nationwide trends in the incidence of all cause and st elevation myocardial infarction associated cardiogenic shock based on gender and race in the United States. Cardiovasc Revasc Med.

[5] Thiele H, Zeymer U, Neumann FJ, Ferenc M, Olbrich HG, Hausleiter J, Richardt G, Hennersdorf M, Empen K, Fuernau G, Desch S, Eitel I, Hambrecht R, Fuhrmann J, Bohm M, Ebelt H, Schneider S, Schuler G, Werdan K (2012). Intraaortic balloon support for myocardial infarction with cardiogenic shock. N Engl J Med.

[6] Burkhoff D, Cohen H, Brunckhorst C, O'Neill WW (2006). A randomized multicenter clinical study to evaluate the safety and efficacy of the tandemheart percutaneous ventricular assist device versus conventional therapy with intraaortic balloon pumping for treatment of cardiogenic shock. Am Heart J.

[7] Seyfarth M, Sibbing D, Bauer I, Frohlich G, Bott-Flugel L, Byrne R, Dirschinger J, Kastrati A, Schomig A (2008). A randomized clinical trial to evaluate the safety and efficacy of a percutaneous left ventricular assist device versus intra-aortic balloon pumping for treatment of cardiogenic shock caused by myocardial infarction. J Am Coll Cardiol.

[8] Schreiber TL, Kodali UR, O'Neill WW, Gangadharan V, Puchrowicz-Ochocki SB, Grines CL (1998). Comparison of acute results of prophylactic intraaortic balloon pumping with cardiopulmonary support for percutaneous transluminal coronary angioplasty (pcta). Catheter Cardiovasc Diagn.

[9] O'Neill WW, Kleiman NS, Moses J, Henriques JP, Dixon S, Massaro J, Palacios I, Maini B, Mulukutla S, Dzavik V, Popma J, Douglas PS, Ohman M (2012). A prospective, randomized clinical trial of hemodynamic support with impella 2.5 versus intra-aortic balloon pump in patients undergoing high-risk percutaneous coronary intervention: The protect ii study. Circulation.

[10] Perera D, Stables R, Clayton T, De Silva K, Lumley M, Clack L, Thomas M, Redwood S (2013). Long-term mortality data from the balloon pump-assisted coronary intervention study (bcis-1): a randomized, controlled trial of elective balloon counterpulsation during high-risk percutaneous coronary intervention. Circulation..

[11] Ye L, Zheng M, Chen Q, Li G, Deng W, Ke D (2014). Effects of intra-aortic balloon counterpulsation pump on mortality of acute myocardial infarction. PLoS One.

[12] Mishra S, Chu WW, Torguson R, Wolfram R, Deible R, Suddath WO, Pichard AD, Satler LF, Kent KM, Waksman R (2006). Role of prophylactic intra-aortic balloon pump in high-risk patients undergoing percutaneous coronary intervention. Am J Cardiol.

[13] Stone GW, Marsalese D, Brodie BR, Griffin JJ, Donohue B, Costantini C, Balestrini C, Wharton T, Esente P, Spain M, Moses J, Nobuyoshi M, Ayres M, Jones D, Mason D, Grines L, O'Neill WW, Grines CL (1997). A prospective, randomized evaluation of prophylactic intraaortic balloon counterpulsation in high risk patients with acute myocardial infarction treated with primary angioplasty. Second primary angioplasty in myocardial infarction (pami-ii) trial investigators. J Am College Cardiol.

[14] Ohman EM, Nanas J, Stomel RJ, Leesar MA, Nielsen DW, O'Dea D, Rogers FJ, Harber D, Hudson MP, Fraulo E, Shaw LK, Lee KL (2005). Thrombolysis and counterpulsation to improve survival in myocardial infarction complicated by hypotension and suspected cardiogenic shock or heart failure: results of the tactics trial. J Thromb Thrombolysis.

[15] O’Gara PT, Kushner FG, Ascheim DD, Casey DE, Chung MK, de Lemos JA, Ettinger SM, Fang JC, Fesmire FM, Franklin BA, Granger CB, Krumholz HM, Linderbaum JA, Morrow DA, Newby LK, Ornato JP, Ou N, Radford MJ, Tamis-Holland JE, Tommaso CL, Tracy CM, Woo YJ, Zhao DX, Anderson JL, Jacobs AK, Halperin JL, Albert NM, Brindis RG, Creager MA, DeMets D, Guyton RA, Hochman JS, Kovacs RJ, Kushner FG, Ohman EM, Stevenson WG, Yancy CW (2013). 2013 accf/aha guideline for the management of st-elevation myocardial infarction: A report of the american college of cardiology foundation/american heart association task force on practice guidelines. Circulation.

[16] Ibanez B, James S, Agewall S, Antunes MJ, Bucciarelli-Ducci C, Bueno H, ALP C, Crea F, Goudevenos JA, Halvorsen S, Hindricks G, Kastrati A, Lenzen MJ, Prescott E, Roffi M, Valgimigli M, Varenhorst C, Vranckx P, Widimsky P (2018). 2017 esc guidelines for the management of acute myocardial infarction in patients presenting with st-segment elevation: The task force for the management of acute myocardial infarction in patients presenting with st-segment elevation of the european society of cardiology (esc). Eur Heart J.

[17] Thygesen K, Alpert JS, Jaffe AS, Simoons ML, Chaitman BR, White HD, Thygesen K, Alpert JS, White HD, Jaffe AS, Katus HA, Apple FS, Lindahl B, Morrow DA, Chaitman BR, Clemmensen PM, Johanson P, Hod H, Underwood R, Bax JJ, Bonow JJ, Pinto F, Gibbons RJ, Fox KA, Atar D, Newby LK, Galvani M, Hamm CW, Uretsky BF, Steg PG, Wijns W, Bassand JP, Menasche P, Ravkilde J, Ohman EM, Antman EM, Wallentin LC, Armstrong PW, Simoons ML, Januzzi JL, Nieminen MS, Gheorghiade M, Filippatos G, Luepker RV, Fortmann SP, Rosamond WD, Levy D, Wood D, Smith SC, Hu D, Lopez-Sendon JL, Robertson RM, Weaver D, Tendera M, Bove AA, Parkhomenko AN, Vasilieva EJ, Mendis S, Bax JJ, Baumgartner H, Ceconi C, Dean V, Deaton C, Fagard R, Funck-Brentano C, Hasdai D, Hoes A, Kirchhof P, Knuuti J, Kolh P, McDonagh T, Moulin C, Popescu BA, Reiner Z, Sechtem U, Sirnes PA, Tendera M, Torbicki A, Vahanian A, Windecker S, Morais J, Aguiar C, Almahmeed W, Arnar DO, Barili F, Bloch KD, Bolger AF, Botker HE, Bozkurt B, Bugiardini R, Cannon C, de Lemos J, Eberli FR, Escobar E, Hlatky M, James S, Kern KB, Moliterno DJ, Mueller C, Neskovic AN, Pieske BM, Schulman SP, Storey RF, Taubert KA, Vranckx P, Wagner DR (2012). Third universal definition of myocardial infarction. J Am Coll Cardiol.

[18] Li J, Li X, Wang Q, Hu S, Wang Y, Masoudi FA, Spertus JA, Krumholz HM, Jiang L (2015). St-segment elevation myocardial infarction in China from 2001 to 2011 (the China peace-retrospective acute myocardial infarction study): a retrospective analysis of hospital data. Lancet..

[19] Song F, Yu M, Yang J, Xu H, Zhao Y, Li W, Wu D, Wang Z, Wang Q, Gao X, Wang Y, Fu R, Sun Y, Gao R, Yang Y (2016). Symptom-onset-to-balloon time, st-segment resolution and in-hospital mortality in patients with st-segment elevation myocardial infarction undergoing primary percutaneous coronary intervention in China: from China acute myocardial infarction registry. Am J Cardiol.

[20] Kolte D, Khera S, Aronow WS, Mujib M, Palaniswamy C, Sule S, Jain D, Gotsis W, Ahmed A, Frishman WH, Fonarow GC (2014). Trends in incidence, management, and outcomes of cardiogenic shock complicating st-elevation myocardial infarction in the United States. J Am Heart Assoc.

[21] Sleeper LA, Ramanathan K, Picard MH, Lejemtel TH, White HD, Dzavik V, Tormey D, Avis NE, Hochman JS (2005). Functional status and quality of life after emergency revascularization for cardiogenic shock complicating acute myocardial infarction. J Am Coll Cardiol.

[22] Vallabhajosyula S, Dunlay SM, Kashani K, Sundaragiri PR, Jaffe AS, Barsness GW (2019). Temporal trends and outcomes of prolonged invasive mechanical ventilation and tracheostomy use in acute myocardial infarction with cardiogenic shock in the United States. Int J Cardiol.

[23] Vallabhajosyula S, Dunlay SM, Murphree DH, Barsness GW, Sandhu GS, Lerman A, Prasad A (2019). Cardiogenic shock in takotsubo cardiomyopathy versus acute myocardial infarction: an 8-year national perspective on clinical characteristics, management, and outcomes. JACC Heart Fail.

[24] Helgestad OKL, Josiassen J, Hassager C, Jensen LO, Holmvang L, Sorensen A, Frydland M, Lassen AT, Udesen NLJ, Schmidt H, Ravn HB, Moller JE (2019). Temporal trends in incidence and patient characteristics in cardiogenic shock following acute myocardial infarction from 2010 to 2017: A danish cohort study. Eur J Heart Fail.

[25] Harjola VP, Lassus J, Sionis A, Kober L, Tarvasmaki T, Spinar J, Parissis J, Banaszewski M, Silva-Cardoso J, Carubelli V, Di Somma S, Tolppanen H, Zeymer U, Thiele H, Nieminen MS, Mebazaa A (2015). Clinical picture and risk prediction of short-term mortality in cardiogenic shock. Eur J Heart Fail.

[26] De Luca G, Savonitto S, Greco C, Parodi G, Dajelli Ermolli NC, Silva C, Lucci D, Gonzini L, Maggioni AP, Cuccia C (2008). Cardiogenic shock developing in the coronary care unit in patients with st-elevation myocardial infarction. J Cardiovasc Med (Hagerstown).

[27] Ndrepepa G, Colleran R, Kastrati A (2017). Reperfusion injury in st-segment elevation myocardial infarction: the final frontier. Coron Artery Dis.

[28] Obling L, Frydland M, Hansen R, Moller-Helgestad OK, Lindholm MG, Holmvang L, Ravn HB, Wiberg S, Thomsen JH, Jensen LO, Kjaergaard J, Moller JE, Hassager C (2018). Risk factors of late cardiogenic shock and mortality in st-segment elevation myocardial infarction patients. Eur Heart J Acute Cardiovasc Care.

[29] Wei Z, Bai J, Dai Q, Wu H, Qiao S, Xu B, Wang L (2018). The value of shock index in prediction of cardiogenic shock developed during primary percutaneous coronary intervention. BMC Cardiovasc Disord.

[30] Abe N, Miura T, Miyashita Y, Hashizume N, Ebisawa S, Motoki H, Tsujimura T, Ishihara T, Uematsu M, Katagiri T, Ishihara R, Tosaka A, Ikeda U (2017). Long-term prognostic implications of the admission shock index in patients with acute myocardial infarction who received percutaneous coronary intervention. Angiology..

[31] Lindholm MG, Kober L, Boesgaard S, Torp-Pedersen C, Aldershvile J (2003). Cardiogenic shock complicating acute myocardial infarction; prognostic impact of early and late shock development. Eur Heart J.

[32] Hasdai D, Topol EJ, Califf RM, Berger PB, Holmes DR (2000). Cardiogenic shock complicating acute coronary syndromes. Lancet..

[33] Hasdai D, Harrington RA, Hochman JS, Califf RM, Battler A, Box JW, Simoons ML, Deckers J, Topol EJ, Holmes DR (2000). Platelet glycoprotein iib/iiia blockade and outcome of cardiogenic shock complicating acute coronary syndromes without persistent st-segment elevation. J Am Coll Cardiol.

[34] Webb JG, Sanborn TA, Sleeper LA, Carere RG, Buller CE, Slater JN, Baran KW, Koller PT, Talley JD, Porway M, Hochman JS (2001). Percutaneous coronary intervention for cardiogenic shock in the shock trial registry. Am Heart J.

[35] Bonnefoy E, Steg PG, Boutitie F, Dubien PY, Lapostolle F, Roncalli J, Dissait F, Vanzetto G, Leizorowicz A, Kirkorian G, Mercier C, McFadden EP, Touboul P (2009). Comparison of primary angioplasty and pre-hospital fibrinolysis in acute myocardial infarction (captim) trial: a 5-year follow-up. Eur Heart J.

